# Hyperthermia and Early Growth of Cerebral Infarct: The Potential Role of Blood–Brain Barrier Permeability

**DOI:** 10.1007/s12975-025-01349-x

**Published:** 2025-04-07

**Authors:** Crhistian-Mario Oblitas, Ana Sampedro-Viana, Sabela Fernández-Rodicio, Manuel Rodríguez-Yáñez, Iria López-Dequidt, Arturo Gonzalez-Quintela, Antonio J. Mosqueira, Jacobo Porto-Álvarez, Javier Martínez Fernández, Marcos Bazarra-Barreiros, María Teresa Abengoza-Bello, Sara Ortega-Espina, Alberto Ouro, Francisco Campos, Tomás Sobrino, José Castillo, Maria Luz Alonso-Alonso, Pablo Hervella, Ramón Iglesias-Rey

**Affiliations:** 1 Neuroimaging and Biotechnology Laboratory (NOBEL), Clinical Neurosciences Research Laboratory (LINC), Health Research Institute of Santiago de Compostela (IDIS), Santiago de Compostela, 15706 A Coruña, Spain; 2 Stroke Unit, Department of Neurology, Hospital Clínico Universitario, Santiago de Compostela, 15706 A Coruña, Spain; 3 Department of Neurology, Hospital Clínico Universitario de Ferrol, Ferrol, 15405 A Coruña, Spain; 4 Department of Internal Medicine, Hospital Clínico Universitario, Santiago de Compostela, 15706 A Coruña, Spain; 5 Department of Neuroradiology, Hospital Clínico Universitario, Health Research Institute of Santiago de Compostela (IDIS), Santiago de Compostela, 15706 A Coruña, Spain; 6 Neuroaging Laboratory Group (NEURAL), Clinical Neurosciences Research Laboratory (LINC), Health Research Institute of Santiago de Compostela (IDIS), Santiago de Compostela, 15706 A Coruña, Spain; 7 Centro de Investigación Biomédica en Red de Enfermedades Neurodegenerativas, Instituto de Salud Carlos III, 28029 Madrid, Spain; 8 Translational Stroke Laboratory (TREAT), Clinical Neurosciences Research Laboratory (LINC), Health Research Institute of Santiago de Compostela (IDIS), Santiago de Compostela, 15706 A Coruña, Spain

**Keywords:** Blood–brain barrier, Early infarct growth, Hyperthermia, Microalbuminuria, sTWEAK

## Abstract

Hyperthermia within the first 24 h following ischemic stroke (IS) has been associated with poor outcomes. We sought to determine whether blood–brain barrier (BBB) permeability contributes to the relationship between hyperthermia and early infarct growth (EIG). A retrospective analysis was conducted on a prospective stroke biobank. EIG was defined as the percentage difference between the initial volume (mL) determined by the diffusion-weighted imaging at admission and the volume (mL) from the control CT image on the 4 th–7 th day. Hyperthermia was defined as an axillary body temperature ≥ 37.5 °C within the first 24 h. Soluble tumor necrosis factor-like weak inducer of apoptosis (sTWEAK) serum levels were measured by ELISA. One-hundred and two (19.7%) patients showed EIG from a cohort of 519 patients (45.6% females). Linear correlation was observed for axillar body temperature and EIG (Pearson’s *r* = 0.46; *p* < 0.001). sTWEAK serum levels showed a *c*-statistic of 0.74 (95% CI: 0.69–0.79), with an optimal cut-off point > 3000 pg/mL for EIG prediction. Moreover, microalbuminuria levels strongly correlated with sTWEAK levels (Pearson’s *r* = 0.75; *p* < 0.001). In the multivariate analysis for EIG was observed an independent association with hyperthermia (adjusted OR 24.21; 95% CI: 12.03–39.12), sTWEAK levels > 3000 pg/mL (adjusted OR 16.43; 95% CI: 3.71–72.70), leukoaraiosis (adjusted OR 10.42; 95% CI: 2.68–39.08), and microalbuminuria (adjusted OR 1.02; 95% CI: 1.00–1.12). In our cohort, hyperthermia was independently associated with EIG after IS. The fact that microalbuminuria, leukoaraiosis, and sTWEAK were also associated with EIG suggests a relationship with increased BBB permeability.

## Introduction

Hyperthermia (body temperature ≥ 37.5 °C) within the first 24 h following ischemic stroke (IS) is relatively common, affecting up to 20–50% of patients, and has a detrimental impact on clinical outcomes [1–3]. Hyperthermia is independently associated with poor outcomes in terms of both morbidity and mortality, especially when it appears within the first 24 h from stroke onset [4–6]. Moreover, in experimental animal models and humans, hyperthermia has been linked to larger cerebral infarct volumes [7–9], contributing to and accelerating necrosis in the brain tissue [4, 5, 9, 10]. However, the pathophysiological mechanisms linking early infarct growth (EIG) and hyperthermia remain not firmly established. Conversely, previous studies have associated elevated levels of soluble TNF-like weak inducer of apoptosis (sTWEAK) to leukoaraiosis, early hematoma growth following intracerebral hemorrhage, and increased blood–brain barrier (BBB) permeability [11–13]. Similarly, microalbuminuria has been associated with hemorrhagic transformation after ischemic stroke (IS) [14–16], suggesting their potential role as surrogate biomarkers for assessing BBB disruption.

The primary aim of this study was to explore whether BBB permeability contributes to the relationship between hyperthermia and EIG after IS. Secondary objectives included evaluating BBB disruption by measuring serum sTWEAK levels, assessing microalbuminuria, and examining the presence of preexisting leukoaraiosis.

## Methods

### Study Design

**Fig. 1:**
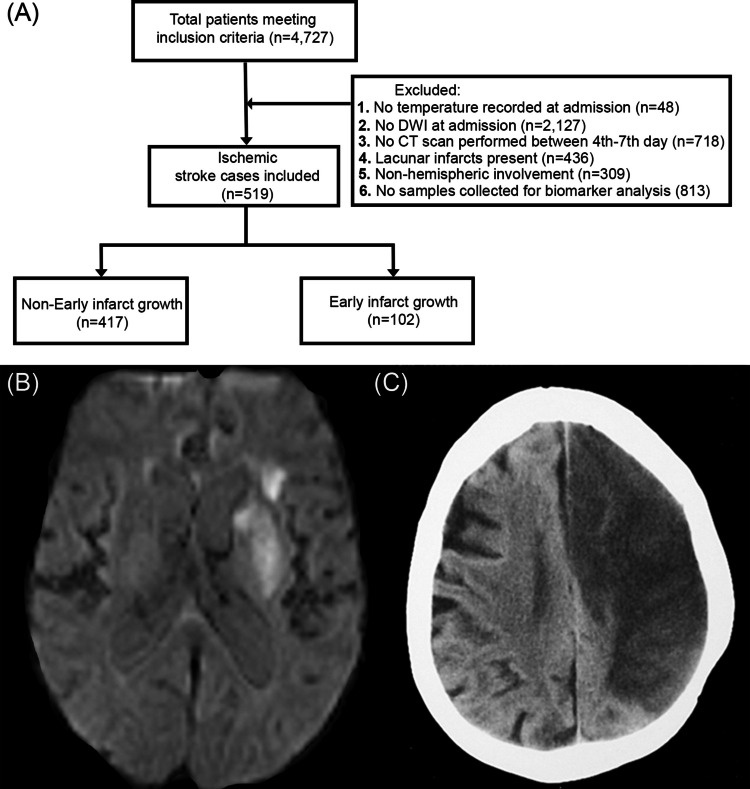
Patient inclusion and exclusion criteria flowchart (**A**). DWI image shows infarct volume on admission (**B**) and the control CT image shows infarct volume on the 4 th–7 th day (**C**). DWI denotes diffusion-weighted imaging; CT denotes computed tomography

The present study included patients older than 18 years with IS events with a known onset time and lasting less than 24 h, admitted to the Stroke Unit at the Hospital Clínico Universitario de Santiago de Compostela (Spain) between January 2012 and December 2018, and prospectively registered in an approved biobank Biobanco Ictus del Complejo Hospitalario Universitario de Santiago (BICHUS). All patients underwent diffusion-weighted imaging (DWI) via magnetic resonance imaging (MRI) upon admission, followed by a control cranial computed tomography (CT) scan between the 4 th and 7 th days after stroke onset. Axillary body temperature was recorded within the first 24 h. The exclusion criteria were (1) absence of diffusion-weighted imaging MRI at diagnosis; (2) absence of follow-up cranial CT between days 4 th–7 th from stroke onset; (3) absence of recorded axillar body temperature within first 24 h; (4) lacunar ischemic strokes; (5) non-hemispheric ischemic strokes; and (6) absence of frozen serum samples for biomarker analysis (Fig. 1).

### Clinical Variables and Neuroimaging Studies

The registry includes demographic variables, vascular risk factors, time from the beginning of the symptomatology until the administration of the reperfusion treatments (fibrinolysis or thrombectomy), comorbidities, associated treatments, axillary body temperature and blood pressure, blood count and coagulation, and biochemical variables. Stroke etiology classification was made using the TOAST (Trial of Org 10,172 in Acute Stroke Treatment) criteria [17]. Neuroimaging included in this research was DWI at admission and CT control between the 4 th and 7 th day, according to the Stroke Unit local protocol. Chronic BBB dysfunction was assessed by the presence and severity of leukoaraiosis using the Fazekas scale with a total score of 0 to 6 (group I, 1–2; group II, 3–4; group III, 5–6) [18]. The IS lesion volumes were determined using the ABC/2 method until 2016 and afterward through the automated planimetric method, according to the Stroke Unit local protocol [19]. Expert neuroradiologists, blinded to clinical data, performed neuroimaging evaluations and classification of leukoaraiosis.

For this research, EIG was defined as the percentage difference between the initial volume (mL) determined by the DWI MRI image at admission and the volume (mL) from the control CT image on the 4 th and 7 th day.

Temperature was taken every 6 h in the dry axilla of patients on admission within 24 h, monitored by the nursing team. For this study, only the highest temperature recorded within the first 24 h from the stroke onset was considered in the data analyses. Hyperthermia was defined as an axillary body temperature ≥ 37.5 °C. In all patients with hyperthermia, blood analyses, chest x-rays, blood cultures for aerobic and anaerobic germs, and sputum and urine cultures were performed for a potential infection, with antipyretic and antimicrobial agents implemented according to the unit protocol. Besides, albuminuria was defined as an albumin-to-creatinine ratio ≥ 30 mg/g creatinine, where microalbuminuria ranges from a ratio between 30 and 300 mg/g and macroalbuminuria for ratios > 300 mg/g creatinine [20].

### Biomarker Determination

Venous blood samples were collected in vacutainer tubes (Becton Dickinson, San Jose, CA, USA) on admission, and centrifuged at 3000 × *g* for 15 min at 4 °C. Serum was immediately aliquoted, frozen, and stored at − 80 °C until analysis. sTWEAK was assessed by experienced researchers in the Clinical Neurosciences Research Laboratory (blinded to clinical and radiological data) using Human sTWEAK enzyme-linked immunosorbent assay (ELISA) kit (Elabscience, USA). Minimum assay sensitivity was 4.69 pg/mL with an intra- and inter-assay CV of 5.06% and 5.21%, respectively. The rest of the biomarkers described were analyzed in the Biochemistry Laboratory of the University Clinical Hospital of Santiago de Compostela.

### Statistical Analysis

This study reported categorical data as proportions and continuous data as mean and one standard deviation (SD) or median and interquartile range (IQR), according to the type of distribution determined by the Kolmogorov–Smirnov test for a sample with the correction of the significance of Lilliefors. The significance of the differences was estimated using Student’s *t*-test or the Mann–Whitney *U* test. One-way analysis of variance (ANOVA) was used to compare differences between more than two groups. The qualitative variables were expressed as percentages, and for the differences, the chi-square test and, if applicable, the uncertainty coefficient. The independent variables associated with EIG were estimated using multiple regression models identifying continuous or categorical variables. First, we carried out logistic regression models, including relevant variables with significant differences according to demographic, clinical, and neuroimaging data. With the selected variables, a new logistic regression model was developed, which finally included the result of the biomarker analysis. To detect the ability of biomarkers to classify the values associated with EIG, ROC (receiver operating characteristic) curves were developed, converting continuous variables into categorical ones for a value that offers maximum sensitivity and specificity. The results were expressed as odds ratio (OR) with 95% confidence intervals (95% CI). Significant values of *p* < 0.05 were considered. The analyses were performed with IBM SPSS v.25 for Mac.

### Standard Protocol Approvals, Registrations, and Patient Consents

This research adheres to good clinical practice guidelines following the Declaration of Helsinki by the World Medical Association (1964) and its subsequent revision in Fortaleza (2013). Both the BICHUS biobank and this research have been approved by the Galician Ethics Committee (project identification code 2019/616). Informed consent was obtained from all patients (or authorized representatives) included in the study, with participant anonymity preserved.

## Results

Among the 519 patients included in the final analysis, 102 patients (19.7%) showed EIG. Overall mean age was 71.7 ± 15 years, 238 females (45.6%) and 281 males (54.1%). According to the TOAST criteria, 38.6% (199 patients) were of cardioembolic origin, 36.1% (187 patients) were of indeterminate origin, and 25.6% (133 patients) were atherothrombotic. IS patients with EIG showed greater severity of NIHSS at admission (13 [10–18] vs. 18 [13–20]; *p* < 0.001), larger DWI volume at admission (27.9 ± 38.7 mL vs. 48.6 ± 43.2 mL; *p* < 0.001), and larger 4 th–7 th day infarct volume (19.4 ± 31.2 vs. 70.9 ± 68.2; *p* < 0.001). There was no statistically significant difference for patients who received or did not receive reperfusion therapy related to EIG (23.5% vs. 31.7%; *p* = 0.07). Further, there were no statistically significant differences (*p* = 0.06) for the association between hyperthermia and EIG according to stroke subtype atherotrombotic (19.7%), cardioembolic (23.2%), and undetermined (21.8%). Table 1 summarizes the results obtained for our cohort related to the presence or not of EIG.

**Table 1:** Baseline characteristics among patients who presented or not early infarct growth (EIG) after an ischemic stroke event

	No EIG *n* = 417	EIG *n* = 102	*p*
Age, years	71.4 ± 12.7	75.7 ± 10.7	0.002
Women, %	46.3	44.1	0.027
Hypertension, %	62.1	63.7	0.428
Diabetes, %	23.3	25.5	0.361
LDL-cholesterol, mg/dL	115.1 ± 38.2	101.5 ± 28.3	0.257
Smoking, %	18.7	18.6	0.556
Atrial fibrillation, %	14.6	28.4	0.001
Axillary temperature, °C	36.3 ± 0.6	37.2 ± 0.9	< 0.001
Hyperthermia, %	2.4	42.7	< 0.001
Blood glucose, mg/dL	147.1 ± 61.8	150.4 ± 60.6	0.319
Leukocytes, × 10^3^/mL	9.4 ± 2.9	9.9 ± 2.6	< 0.001
Fibrinogen, mg/dL	451.2 ± 99.9	438.4 ± 80.3	0.006
C-reactive protein, mg/dL	2.8 ± 3.5	3.9 ± 2.9	< 0.001
Microalbuminuria, mg/g 24 h	47.1 ± 63.0	124.2 ± 75.7	< 0.001
sTWEAK, pg/mL	3385.9 ± 2014.2	4861.3 ± 1670.6	0.006
sTWEAK > 3000 pg/mL, %	50.6	91.2	< 0.001
NIHSS at admission	13 [10, 18]	18 [12, 20]	< 0.001
mRS at 3 months	2 [1, 3]	4 [2, 5]	< 0.001
DWI on admission, mL	27.9 ± 38.7	48.6 ± 43.2	< 0.001
CT 4 th–7 th day, mL	19.4 ± 31.2	70.9 ± 68.2	< 0.001
TOAST			0.011
Cardioembolic, %	36.5	46.1	
Atherothrombotic, %	27.3	18.6	
Undeterminate, %	36.2	35.3	
Leukoaraiosis, %	14.4	86.6	< 0.001
Degree of leukoaraiosis, %			< 0.001
No	85.6	13.4	
Fazekas I	11.6	36.1	
Fazekas II	2.3	32.0	
Fazekas III	0.5	18.6	
Reperfusion therapy, %	31.7	23.5	0.065

*CT*, computed tomography; *DWI*, diffusion-weighted imaging; *mRS*, Rankin scale modified; *NIHSS*, National Institutes of Health Stroke Scale; *sTWEAK*, soluble tumor necrosis factor-like weak inducer of apoptosis

### Association of Hyperthermia, EIG, and Acute BBB Permeability

**Fig. 2:**
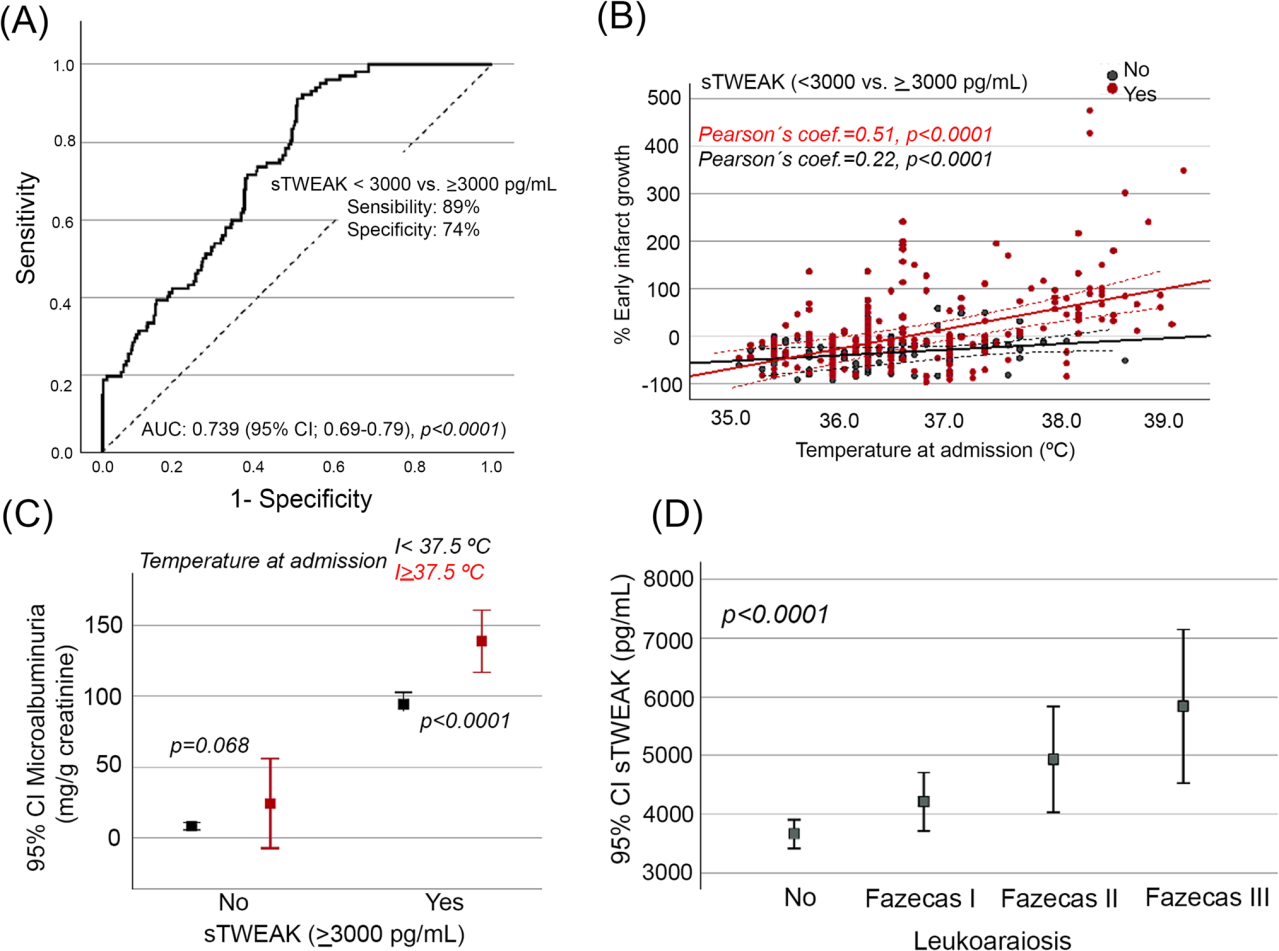
Area under the ROC curve analysis for early infarct growth prediction capability of sTWEAK showed a *c*-statistic of 0.74 (95% CI: 0.69–0.79; *p* < 0.001) with an optimal cut-off point > 3000 pg/mL (**A**). The correlation between early infarct growth and hyperthermia was significantly stronger (Pearson’s *r* = 0.51; *p* < 0.001) when sTWEAK levels were higher (sTWEAK > 3000 pg/mL) (**B**). Microalbuminuria and hyperthermia were significantly correlated when sTWEAK levels were high > 3000 pg/mL (*p* < 0.001) (**C**). Association for sTWEAK levels and the presence and severity of leukoaraiosis (**D**). sTWEAK denotes soluble tumor necrosis factor-like weak inducer of apoptosis. sTWEAK denotes soluble tumor necrosis factor-like weak inducer of apoptosis

EIG was associated with higher axillary body temperature (37.2 ± 0.9 vs. 36.3 ± 0.6; *p* < 0.001), finding a linear correlation for axillary body temperature and EIG (Pearson’s *r* = 0.46; *p* < 0.001). Further, hyperthermia was more frequent in patients with EIG (42.7% vs. 2.4%; *p* < 0.001). The assessment of microalbuminuria and serum sTWEAK levels indirectly evaluated acute BBB permeability. In this setting, IS patients who developed EIG showed higher mean values of microalbuminuria (124.2 mg/g vs. 47.1 mg/g; *p* < 0.001) and sTWEAK levels (4861.3 pg/mL vs. 3,385.9 pg/mL; *p* < 0.001) (Table 1); however, microalbuminuria showed a strong correlation with sTWEAK levels (Pearson’s *r* = 0.75; *p* < 0.001). Further, sTWEAK showed a *c*-statistic of 0.74 (95% CI: 0.69–0.79; *p* < 0.001) for prediction of EIG and an optimal cut-off point ≥ 3000 pg/mL (sensitivity 89% and specificity 74%) (Fig. 2A). Thus, the correlation between EIG and axillary body temperature showed a moderate correlation when sTWEAK ≥ 3000 pg/mL (Pearson’s *r* = 0.51; *p* < 0.001) compared with sTWEAK < 3000 pg/mL (Pearson’s *r* = 0.22; *p* < 0.001) (Fig. 2B). On the other hand, the relationship between microalbuminuria and axillar body temperature was statistically significant only when sTWEAK ≥ 3000 pg/mL (*p* < 0.001) (Fig. 2C).

### Chronic BBB Dysfunction and Hyperthermia for EIG

Chronic BBB dysfunction was assessed by the presence and severity of leukoaraiosis, which was more frequent in IS patients with EIG (86.6% vs. 14.4%; *p* < 0.001). The relationship of EIG was stronger according to the severity of leukoaraiosis (43.5% for Fazekas I vs. 90.5% for Fazekas III). When evaluating the linear correlation between axillary body temperature and EIG considering the presence of leukoaraiosis, it was observed: Fazekas I (Pearson’s *r* = 0.39; *p* < 0.01), Fazekas II (Pearson’s *r* = 0.63; *p* < 0.01), and Fazekas III (Pearson’s *r* = 0.73; *p* < 0.01) (Fig. 2D).

### Binary and Multivariable Analysis for EIG

Binary logistic regression analysis included relevant clinical and biomarker variables for patients with and without EIG. Those who showed a statistically significant association were included in the multivariate analysis. After adjustment in the multivariable logistic regression analysis, independent association for EIG was observed only for hyperthermia (adjusted OR 24.21; 95% CI: 12.03–39.12), sTWEAK levels > 3000 pg/mL (adjusted OR 16.43; 95% CI: 3.71–72.70), leukoaraiosis (adjusted OR 10.42; 95% CI: 2.68–39.08), and microalbuminuria (adjusted OR 1.02; 95% CI: 1.00–1.12). Table 2 summarizes the findings of the binary and multivariable logistic regression analyses.

**Table 2:** Binary and multivariable logistic regression analysis for early infarct growth (dependent variable)

	Bivariate			Multivariate		
	OR	CI 95%	*p*	aOR*	CI 95%	*p*
Age (years)	1.03	1.00–1.04	0.004	1.04	0.98–1.12	0.146
Sex (women)	1.65	1.07–2.56	< 0.001	1.31	0.42–4.07	0.660
Atrial fibrillation	2.31	1.39–3.86	0.001	2.14	0.64–8.32	0.291
Hyperthermia	29.71	14.14–62.19	< 0.001	24.21	12.03–39.12	< 0.001
Leukocytes (× 10^3^/mL)	1.15	1.08–1.23	< 0.001	0.90	0.65–2.11	0.416
Fibrinogen (mg/dL)	1.00	1.00–1.01	0.013	1.00	0.99–1.00	0.453
Microalbuminuria (mg/g 24 h)	1.02	1.01–1.03	< 0.001	1.02	1.00–1.12	0.016
C-reactive protein (mg/dL)	1.15	1.08–1.23	< 0.001	0.91	0.76–1.45	0.510
DWI volume^†^ (mL)	1.00	1.00–1.01	< 0.001	0.99	0.98–1.00	0.511
Leukoaraiosis	38.31	1.00–59.07	< 0.001	10.42	2.68–39.08	< 0.001
NIHSS^†^	1.15	1.10–1.20	< 0.001	1.02	0.85–1.39	0.236
sTWEAK (> 3000 pg/mL)	10.08	4.95–20.53	< 0.001	16.43	3.71–72.70	< 0.001

^*^Adjusted odds ratio for multivariate analysis using variables from bivariate analysis

^†^Measured on admission

*DWI*, diffusion-weighted imaging; *NIHSS*, National Institutes of Health Stroke Scale; *sTWEAK*, soluble tumor necrosis factor-like weak inducer of apoptosis

## Discussion

BBB is crucial for maintaining cerebral homeostasis; however, a hypoxic-metabolic and pro-inflammatory response is triggered, leading to BBB disruption and increased permeability after a stroke event [21, 22]. Recent studies with animal models suggested a continuous and dynamic progression instead of biphasic evolution on permeability in BBB that persists over days to weeks [23, 24]. In a retrospective study, 42 patients with IS who underwent dynamic contrast-enhanced MRI at admission were analyzed for indirect and non-invasive assessment of BBB permeability by comparing contrast agent leakage between the affected region and its contralateral homologous region. Although this was not a longitudinal individual imaging study of stroke evolution, the authors found that while the non-infarcted contralateral areas showed no significant changes over time, the BBB permeability in the infarcted region exhibited a continuous and sustained increase pattern time-dependent [23].

The impact of temperature variation on BBB permeability has been previously evaluated in animal models [25]. Specifically, Kiyatkin et al. [26] examined the effects of temperature per se in BBB permeability inducing hyper- and hypothermia. Their findings showed that the induction of hyperthermia leads to astrocyte activation, accompanied by an increase in water and ion content, consistent with developing edema and temperature-dependent morphological changes in different brain structures. In contrast, hypothermia induction resulted in reduced water and ion levels, indicative of dehydration. These results highlighted the key role of temperature as a regulator of BBB permeability. In this setting, several clinical studies have identified hyperthermia as an independent risk factor associated with larger infarct volumes, greater disability, and mortality after a stroke event, particularly when early hyperthermia occurs with a threshold of ≥ 37.5 °C [10, 22]. Similarly, in cases of intracerebral hemorrhage with ventricular extension, the risk of developing hyperthermia is notably higher [27].

Following the onset of a stroke, a neuroinflammatory response is activated to repair and clear necrotic brain tissue, directly impacting brain architecture and leading to BBB disruption [12, 28, 29]. However, an excessive inflammatory response can exacerbate oxidative stress as microglial and immune cells activate various inflammatory pathways. One such pathway involves sTWEAK, a biomarker of endothelial dysfunction from the TNF superfamily, which is highly expressed in the endothelial cells and perivascular structures of the BBB. Its overexpression ultimately contributes to detrimental effects [28]. Preclinical studies have demonstrated elevated TWEAK expression across the infarcted area in stroke animal models [30–32]. This study assessed acute BBB permeability by evaluating biomarkers such as sTWEAK and microalbuminuria to explore a potential association between hyperthermia and EIG in a clinical setting following an IS. It was observed that nearly 20% of patients developed EIG, showing a linear correlation with the presence of hyperthermia. Further, the higher the sTWEAK levels (above 3000 pg/mL), the stronger the correlation was for hyperthermia and EIG. Additionally, previous studies have suggested that microalbuminuria was independently associated with an increased overall risk of hemorrhagic transformation following IS (adjusted OR 7.45; 95% CI: 2.30–24.16) and intraparenchymal bleeding (adjusted OR 8.30; 95% CI: 1.77–38.89), pointing its potential role as a BBB permeability biomarker [16]. Additionally, a study of 160 IS patients found microalbuminuria in one-third of cases, with a correlation to infarct severity at admission and 3 months later [14]. Another study involving 60 patients after their first IS reported a strong correlation between microalbuminuria and body temperature (Pearson’s *r* = 0.45; *p* < 0.01), with both factors independently linked to poor prognosis. The present study aligns with prior research, while microalbuminuria evaluated in this cohort showed a strong correlation with sTWEAK levels. Moreover, both hyperthermia and sTWEAK > 3000 pg/mL were independently associated with up to 24-fold times (*p* < 0.001) and 16-fold times (*p* < 0.001) increased risk for EIG, respectively, after adjusting by age, sex, diabetes, infarct volume at admission, and NIHSS at admission.

Furthermore, differentiating between the area of irreversible damage due to necrosis, also called the “core,” and the ischemic “penumbra,” which represents severely hypoperfused and hypoxic brain tissue that is at risk but potentially salvageable if blood flow is restored, may be crucial for developing future therapeutic strategies [1, 22, 33, 34]. It is estimated that approximately 20% of patients experience rapid core growth (> 15 mL/h) following stroke onset, 30% exhibit moderate core growth (3–10 mL/h), and up to 50% demonstrate resilient slow core growth (< 1.5 mL/h) [35]. The final size of the core is typically reached within 3–7 days after the ischemic event. Current reperfusion therapies have extended the therapeutic window to 24 h after the onset of symptoms [35–37]. However, despite achieving successful recanalization rates exceeding 80% through pharmacological fibrinolysis and mechanical thrombectomy, up to half of the patients still fail to achieve functional independence, as indicated by a modified Rankin Scale score of less than 2 [22, 38].

Additionally, the presence of white matter lesions, known as leukoaraiosis, identified through neuroimaging, has been associated with various forms of cerebral vascular degeneration. It is considered a potential marker of chronic BBB dysfunction and is linked to an increased risk of both ischemic and hemorrhagic stroke [39]. However, the pathogenesis of leukoaraiosis remains poorly understood, with theories suggesting it may reflect impaired collateral vessel health or a diminished capacity for brain injury repair [40], along with other age-related factors that contribute to endothelial dysfunction and BBB breakdown [13]. In this context, our research group previously identified a connection between the presence and severity of leukoaraiosis and elevated sTWEAK levels [41], suggesting sTWEAK as a potential co-adjuvant therapeutic target for a novel approach to stroke management [42, 43]. Preclinical studies have shown that blocking TWEAK activity before an ischemic event significantly reduces core volume by 30–60%, indicating that sTWEAK may be a promising biomarker for limiting EIG [29, 44, 45]. Based on the findings of this study, our research group hypothesizes that hyperthermia in patients with pre-existing BBB lesions, such as leukoaraiosis, may promote the development of EIG following a stroke. sTWEAK could serve as an indirect marker for assessing BBB disruption, and targeting this pathway may help restore BBB integrity, potentially “freezing” infarct growth and preserving at-risk brain tissue for better functional outcomes. Further preclinical and clinical studies are needed to confirm this hypothesis. Our study has several limitations. First, it is a retrospective, single-center study based on a prospective registry. Second, we used the ABC/2 method to determine the volume of vascular lesions. Previous studies [46] have used an expansion of > 33% as a minimum criterion for determining lesion growth. In our analysis, we have used the percentage of lesion growth between the baseline neuroimaging and that obtained between the 4 th and 7 th day. The ABC/2 method was carried out until 2016, and since then, the volume has been determined by an automated planimetric method, which reduces the error of manual measurement. Due to the need for biological samples, only 37 patients (5.3% of the analyzed sample) had been admitted before 2016. Third, in cerebral infarction, DWI does not exclusively represent the area of cerebral necrosis but rather reflects the presence of intraneuronal edema, which often leads to cell destruction. Likewise, brain CT imaging makes it challenging to differentiate the infarct area with varying degrees of vasogenic edema. Fourth, axillary body temperature was used to assess hyperthermia, which may be less accurate than rectal or tympanic measurements. Finally, direct evaluation of BBB permeability, such as dynamic contrast-enhanced MRI, was not performed. However, the study also has strengths. First, despite being retrospective, it does not present selection biases that could skew the analysis hypothesis, and the sample size permits valid conclusions. Second, there is a plausible pathophysiological explanation linking hyperthermia, elevated sTWEAK levels, and microalbuminuria with the risk of BBB dysfunction. Finally, this study represents translational research, where findings observed in animal models have been tested in a clinical context, with consistent results that open new lines for future research.

## Conclusion

In our cohort, hyperthermia was independently associated with EIG after IS. suggesting a relationship with increased BBB permeability. Notably, higher sTWEAK levels correlated with a greater percentage of EIG. Additionally, leukoaraiosis appeared to amplify the impact of hyperthermia on EIG. Further studies are needed to confirm these findings.

## Data Availability

No datasets were generated or analysed during the current study.
